# Wash-free, label-free immunoassay for rapid electrochemical detection of *Pf*HRP2 in whole blood samples

**DOI:** 10.1038/s41598-018-35471-8

**Published:** 2018-11-20

**Authors:** Gorachand Dutta, Peter B. Lillehoj

**Affiliations:** 1 Department of Electronic and Electrical Engineering, University of Bath, Claverton Down, Bath BA2 7AY UK; 2 Centre for Biosensors, Bioelectronics and Biodevices, University of Bath, Claverton Down, Bath BA2 7AY UK; 3 Department of Mechanical Engineering, Michigan State University, East Lansing, MI 48824 USA; 4 Department of Biomedical Engineering, Michigan State University, East Lansing, MI 48824 USA

## Abstract

Currently, the diagnosis of many diseases relies on laboratory-based immunoassays (ELISA, Western Blot), which are laborious, time-consuming and expensive. To address these limitations, we report a wash-free and label-free electrochemical immunoassay for rapid measurements of protein biomarkers in blood samples. This immunosensor employs a unique detection scheme based on electrochemical-chemical (EC) redox cycling for signal amplification combined with an affinity-based protein quantification strategy. All of the reagents required for this assay are dried and stored on a stacked membrane assembly, consisting of a Vivid Plasma Separation membrane and two cellulose membranes situated above the sensor, enabling excellent stability at room temperature for up to 2 months. Proof of concept was carried out by performing measurements of *Plasmodium falciparum* histidine-rich protein 2 (*Pf*HRP2) in whole blood samples, which could be detected from 100 ng/mL to 100 µg/mL with excellent specificity and reproducibility. Each measurement requires only two liquid dispensing steps and can completed in 5 min, making this diagnostic platform promising for point-of-care testing in resource-limited settings.

## Introduction

Clinical diagnostic tests play an important role in medical care. Many of these tests are based on laboratory-based immunoassays, such as enzyme-linked immunosorbent assay (ELISA) or Western Blot, which are laborious, time-consuming and expensive. To address these limitations, researchers have been developing miniature biosensors which offer enhanced simplicity, faster measurement times and lower costs compared with benchtop diagnostic assays^1,2^. Of all biosensing modalities, electrochemical detection is one of the most popular due to its excellent analytical performance, simplicity, portability and low costs^3,4^. These features also make electrochemical sensors well suited for point-of-care applications, such as health monitoring (e.g. glucose testing) and *in vitro* diagnostic testing in resource-limited settings. Electrochemical immunosensors have been reported for the detection of various disease biomarkers, including carcinoembryonic antigen (CEA)^5–9^, alpha fetoprotein (AFP)^8–10^, *Plasmodium falciparum* histidine-rich protein 2 (*Pf*HRP2)^11^ and prostate specific antigen (PSA)^12–14^. While these devices are promising, many of them require protein labeling and/or washing, which complicates and lengthens the testing process. Additionally, most immunoassays utilize enzymes for signal transduction or amplification, which results in limited stability at room temperature^15^.

Towards a label-free electrochemical assay, Wang *et al*. employed graphene nanocomposite-modified electrodes for measurements of AFP, which could be detected from 10 fg/mL to 10 ng/mL^16^. Alternatively, Wang *et al*. reported an electrochemical sensor based on a poly(thionine)-Au composite electrode for the detection of cytokeratin antigen 21-1, which could be detected from 10 fg/mL to 100 ng/mL^17^. While both of these label-free sensors exhibit high sensitivity and wide detection ranges, they involve complicated sensor modification protocols and washing steps. To address these limitations, Yang’s group developed wash-free electrochemical immunosensors based on proximity-dependent electron mediation of an enzymatic mediator for the detection of cardiac troponin I and PSA^18,19^. While these sensors can detect clinically relevant proteins at pg/mL levels without washing, they use enzyme labels which limits their stability at room temperature.

In this work, we present a wash-free and label-free immunoassay for rapid measurements of protein biomarkers. This nonenzymatic sensor utilizes Ru(NH_3_)_6_^3+^ as an electron mediator, methylene blue (MB) as the mediator substrate, and electrochemical-chemical (EC) redox cycling for rapid (5 min) detection in whole blood samples. Protein quantification is achieved using an affinity-based sensing scheme where surface attachment of the target antigen effectively forms an insulator layer which impedes electron transfer and diminishes the electrochemical signal. In the absence of the target protein, electron transfer via EC redox cycling occurs readily between the redox species and the electrode surface, generating a large electrochemical current. This immunoassay was used for quantitative measurements of *Pf*HRP2, an important malaria biomarker, in whole blood samples. *Pf*HRP2 levels in blood/plasma have been shown to be closely correlated with disease severity, making it a useful diagnostic indicator for *P*. *falciparum* infection^20–22^. *Pf*HRP2 levels in plasma were also found to be useful in predicting when children with uncomplicated malaria progressed to cerebral malaria^23^. While the mean *Pf*HRP2 concentrations for different disease states vary in literature due to differences in the experimental methods or statistical analysis, *Pf*HRP2 levels in individuals with severe *P*. *falciparum* infection and cerebral malaria are significantly higher (~5–10×) than in individuals with uncomplicated malaria, and can range from hundreds of ng/mL to 90,000 ng/mL. Current methods for quantifying *Pf*HRP2 include ELISA^24–26^ and Western Blot^27,28^, which require lengthy and laborious sample processing (e.g. centrifugation, dilution). Malaria rapid diagnostic tests (RDTs) for *Pf*HRP2 are available^29–31^; however these assays only provide qualitative results and are not useful for distinguishing different states of infection. Using this immunoassay, *Pf*HRP2 could be quantified from 100 ng/mL to 100 μg/mL in whole blood samples without sample processing, labeling or washing. In addition to its simplicity and capacity for measuring *Pf*HRP2 at clinically relevant concentrations, this assay exhibits excellent reproducibility, specificity and stability at room temperature, making it well suited for point-of-care testing in developing countries.

## Results and Discussion

### EC redox cycling for wash-free, label-free immunosensing

**Figure 1:**
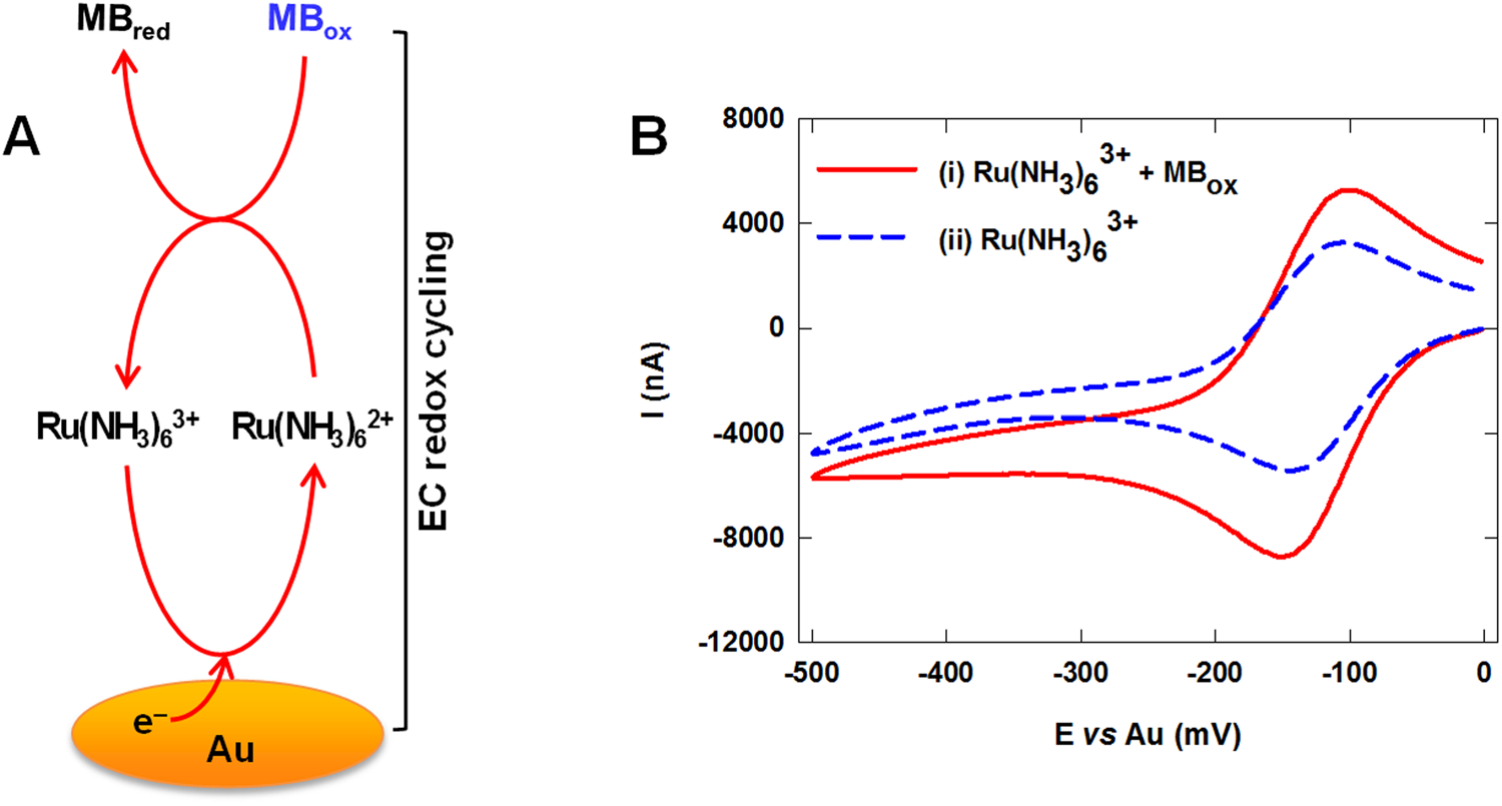
(**A**) Schematic illustration of EC redox cycling with methylene blue (MB) and Ru(NH_3_)_6_^3+^. (**B**) Cyclic voltammograms of PBS solutions containing (i) 1 mM of Ru(NH_3_)_6_^3+^ and 50 µM of MB_ox_, or (ii) 1 mM of Ru(NH_3_)_6_^3+^.

This immunosensor employs a unique EC redox cycling scheme for signal amplification. As shown in Fig. 1A, Ru(NH_3_)_6_^3+^ is reduced to Ru(NH_3_)_6_^2+^ via an electrochemical reaction with the Au electrode (initiated by a bias potential), which is subsequently reoxidized back to Ru(NH_3_)_6_^3+^ by methylene blue via a chemical reaction. This redox cycling process occurs continuously due to the simultaneous regeneration of Ru(NH_3_)_6_^3+^, resulting in a large electrochemical signal. The formal potential of the MB_ox_/MB_red_ couple (−0.12 V) is similar to that of the Ru(NH_3_)_6_^3+^/Ru(NH_3_)_6_^2+^ couple, enabling fast electron transfer to occur between these two couples. Furthermore, the formal potentials of the Ru(NH_3_)_6_^3+^/Ru(NH_3_)_6_^2+^ and MB_ox_/MB_red_ couples are negative, enabling a low bias potential (−0.35 V) to be used, which minimizes the likelihood of interference effects due to electroactive species in blood. In addition to their fast electrokinetics, Ru(NH_3_)_6_^3+^ and methylene blue exhibit excellent stability at room temperature, circumventing the need for refrigeration. To evaluate the effectiveness of this redox cycling scheme, cyclic voltammograms of buffer solutions containing only Ru(NH_3_)_6_^3+^ or both Ru(NH_3_)_6_^3+^ and methylene blue were obtained using bare Au electrodes. As shown in Fig. 1B, the solution containing both Ru(NH_3_)_6_^3+^ and methylene blue (curve i of Fig. 1B) generated a large cathodic current due to the electro-reduction of Ru(NH_3_)_6_^3+^. In contrast, the cyclic voltammogram generated from the solution containing only Ru(NH_3_)_6_^3+^ (curve ii of Fig. 1B) generated a ~2× smaller cathodic current, demonstrating that EC redox cycling between Ru(NH_3_)_6_^3+^ and methylene blue was effective in amplifying the detection signal.

**Figure 2:**
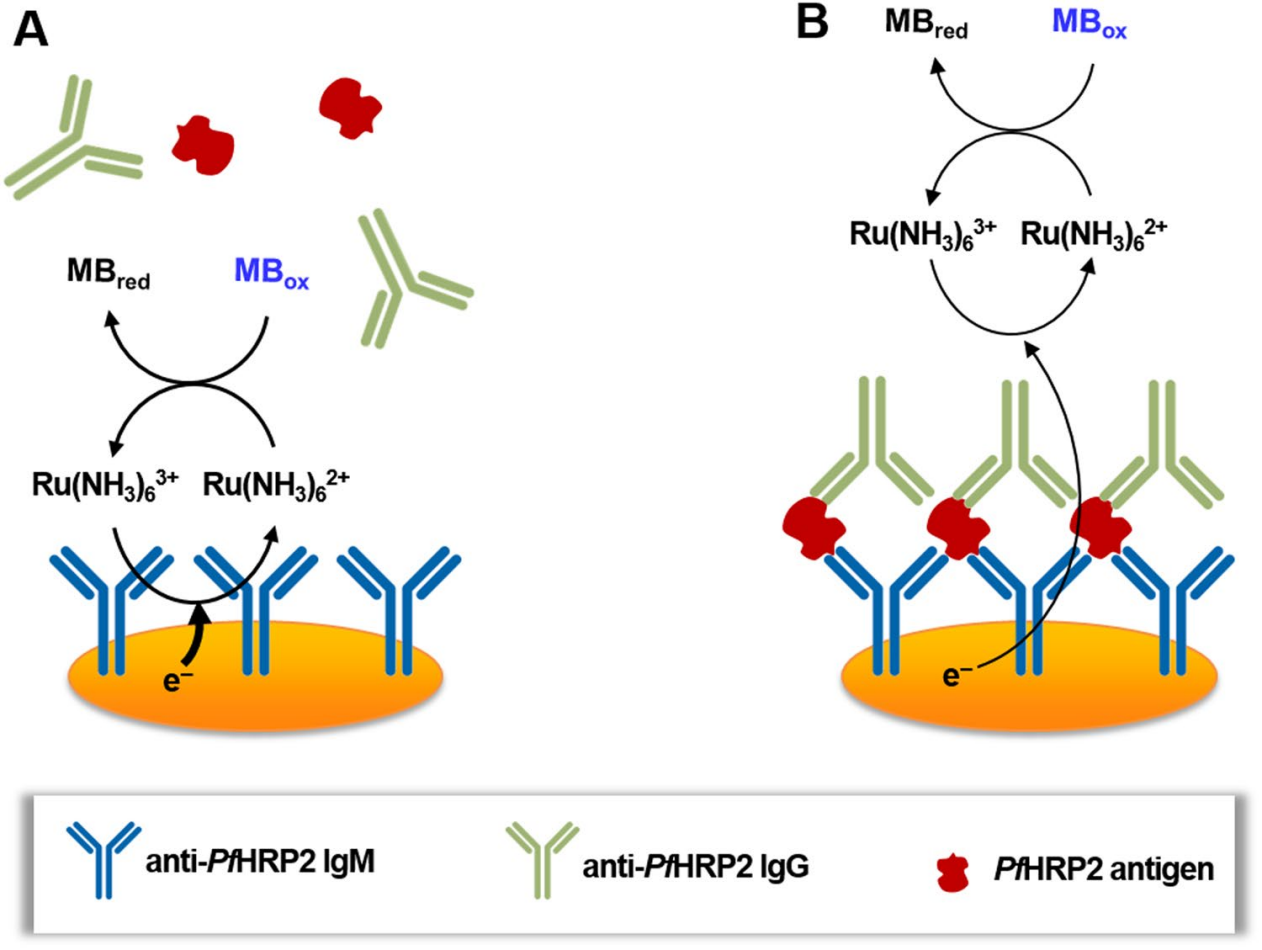
Schematic illustration of the wash-free, label-free electrochemical detection scheme. (**A**) In the absence of the target antigen, a large electrochemical signal is generated due to fast EC redox cycling between the redox species and the electrode surface. (**B**) In the presence of the target antigen, an insulator layer comprised of the antigen-antibody complex is formed on the electrode surface, which hinders electron transfer to the sensor surface, thereby diminishing the electrochemical signal.

To achieve wash-free and label-free protein detection, this EC redox cycling scheme was combined with an immunosensing strategy based on surface accessibility for electron transfer. A schematic illustration of this detection scheme is shown in Fig. 2. In the absence of the target antigen, the redox species can readily access the electrode surface, resulting in fast electron transfer and a large electrochemical current (Fig. 2A). When the target antigen is present in the sample, it binds to the antibody-immobilized electrode and allows subsequent attachment of the secondary anti-*Pf*HRP2 antibody to the electrode surface (Fig. 2B). This protein complex forms an insulator layer which hinders access of the redox species to the electrode surface, limiting the rate of electron transfer. The reduction in the detection signal is inversely proportional to the amount of antigen bound to the electrode surface, which is representative of its concentration in the sample. By optimizing various assay parameters, such as the concentrations of Ru(NH_3_)_6_^3+^, methylene blue and anti-*Pf*HRP2 antibodies, and the applied bias potential, this sensing scheme can detect high protein concentrations (up to 100 µg/mL) without labeling, washing or sample processing (i.e. dilution), which cannot be achieved with prior electrochemical immunosensors^11,16–19^.

**Figure 3:**
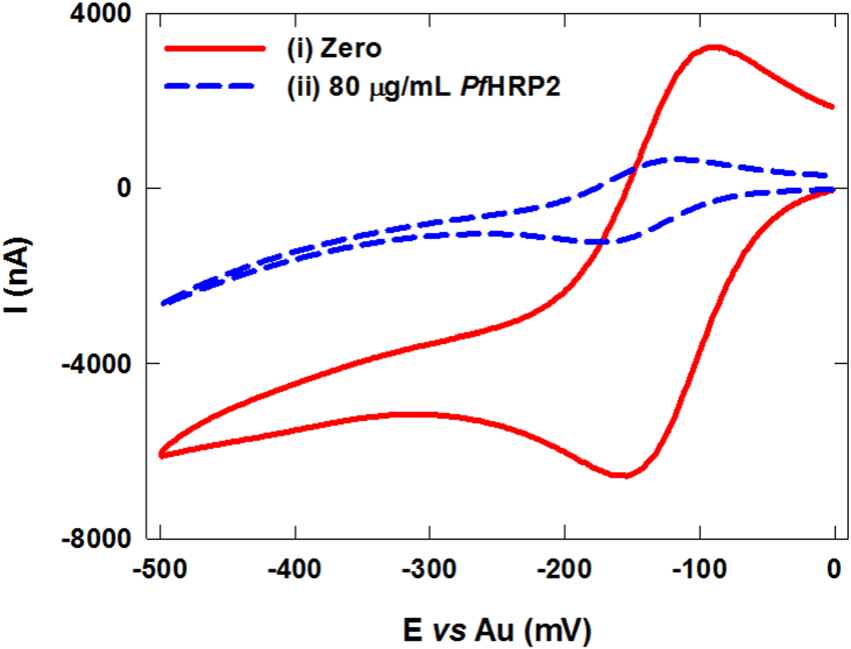
Cyclic voltammograms of non-spiked blood samples (i) and blood samples spiked with 80 µg/mL of *Pf*HRP2 (ii) using the assembled immunosensor.

To validate this detection scheme, cyclic voltammetric measurements were performed using blood samples spiked with 80 µg/mL of *Pf*HRP2 and non-spiked blood samples. As shown in curve i of Fig. 3, a substantial voltammetric current is generated from the blood sample without *Pf*HRP2 due to fast EC redox cycling between the redox species and the electrode surface. In contrast, the blood sample containing 80 µg/mL of *Pf*HRP2 resulted in a significantly lower voltammetric current due to the formation of the protein complex insulator layer on the electrode surface (curve ii in Fig. 3).

### Sensor design and operation

**Figure 4:**
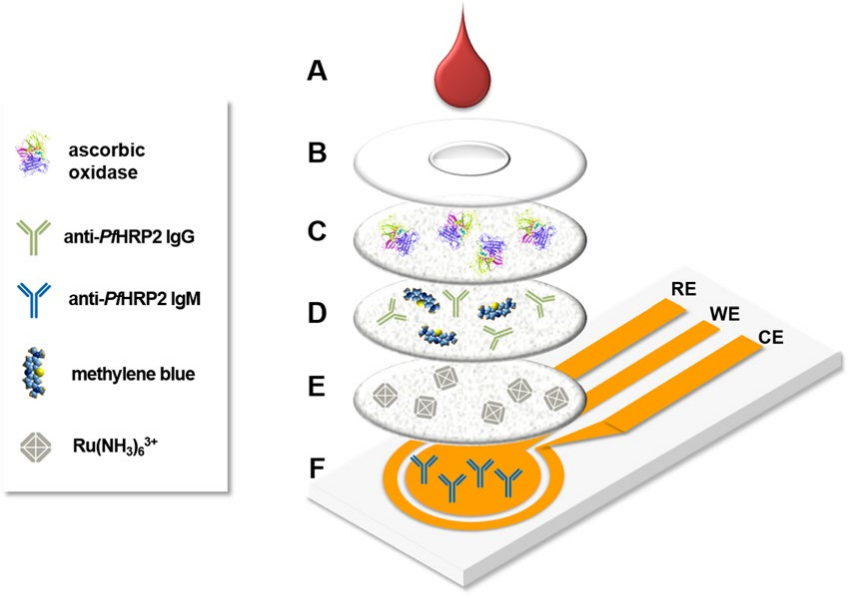
Exploded view of the assembled immunosensor highlighting the major components. (**A**) blood sample; (**B**) PET film with inlet; (**C**) Vivid Plasma Separation membrane containing ascorbic oxidase; (**D**) cellulose membrane containing anti-*Pf*HRP2 secondary antibody and methylene blue; (**E**) cellulose membrane containing Ru(NH_3_)_6_^3+^; (**F**) Au electrochemical sensor (WE: working electrode, CE: counter electrode, and RE: reference electrode).

This immunosensor consists of a tri-layer stacked membrane assembly, containing dried reagents, placed on top of an Au electrochemical sensor (Fig. 4). A hydrophobic PET film with a through-hole is attached to the top of the membrane assembly to prevent lateral spreading of the liquid sample and direct its passage to the underlying membranes. To initiate a measurement, 10 µL of blood is applied to the sensor followed by 30 µL of PBS, which facilitates reconstitution of the dried reagents and subsequent transport through the membrane assembly. As the blood sample encounters the Vivid Plasma Separation membrane, the blood cells and platelets are trapped in the membrane while the plasma passes through. Ascorbic acid in the blood reacts with the AO in the Vivid membrane, preventing it from causing interference during electrochemical detection. The secondary antibody (anti-*Pf*HRP2 IgG), methylene blue, and Ru(NH_3_)_6_^3+^, are reconstituted as the plasma-PBS sample flows through the second and third membranes, which are subsequently transported to the electrode surface by capillary flow.

### Optimization of assay parameters

Several parameters, including the concentrations of Ru(NH_3_)_6_^3+^ and methylene blue, the applied bias potential, and concentrations of primary and secondary anti-*Pf*HRP2 antibodies were optimized to enhance the sensitivity, dynamic range and reproducibility of this assay for *Pf*HRP2 detection in whole blood. Chronocoulometric measurements were first carried out using sensors containing varying concentration of Ru(NH_3_)_6_^3+^ and methylene blue. Background-to-signal ratios (BSRs) were calculated from chronocoulometric charges at 60 sec of chronocoulograms of blood samples containing zero or 50 µg/mL of *Pf*HRP2. The highest BSRs were obtained with sensors containing 1 mM of Ru(NH_3_)_6_^3+^ (Supplementary Fig. S1) and 50 µM of methylene blue (Supplementary Fig. S2). Using these optimized parameters, measurements were performed by applying varying bias potentials from −0.15 to −0.45 V. As shown in Supplementary Fig. S3, the chronocoulometric signals increased steadily with higher bias potentials. While the highest BSR was obtained at −0.45 V, the standard deviations of the background and detection signals were significantly larger than those obtained at lower potentials due to oxygen reduction interference. Therefore, a potential of −0.35 V was selected to minimize potential interference effects. The concentration of secondary anti-*Pf*HRP2 antibody was optimized using chronocoulometric signals of blood samples with and without 50 µg/mL of *Pf*HRP2 containing varying concentration of secondary anti-*Pf*HRP2 antibody (Supplementary Fig. S4). While the background signals of all samples not containing *Pf*HRP2 were similar, the chronocoulometric signals of blood samples containing 50 µg/mL of *Pf*HRP2 steadily decreased with increasing antibody concentrations. At 100 µg/mL, the detection signal leveled off and the BSR decreased at 1,000 µg/mL due to the electrode surface becoming fully saturated with antibody. Based on these results, 100 µg/mL was selected as the optimal concentration for the secondary anti-*Pf*HRP2 antibody. The last parameter that was optimized was the concentration of primary anti-*Pf*HRP2 antibody immobilized on the sensor. Chronocoulometric signals of blood samples with and without 50 µg/mL of *Pf*HRP2 containing 1 mM Ru(NH_3_)_6_^3+^, 50 µM of methylene blue, and 100 µg/mL of secondary anti-*Pf*HRP2 antibody were obtained from sensors immobilized with 1 µg/mL, 10 µg/mL or 100 µg/mL of primary anti-*Pf*HRP2 antibody (Supplementary Fig. S5). The sensors containing 1 µg/mL of primary antibody exhibited the lowest BSR, whereas sensors immobilized with 10 µg/mL or 100 µg/mL of primary antibody exhibited ~2× higher BSRs. While the detection signals from sensors immobilized with 100 µg/mL of primary antibody offered better reproducibility (i.e. smaller standard deviations) than sensors containing 10 µg/mL of primary antibody, there was not a significant improvement in the BSR, which suggests that the sensor surface was saturated with primary antibody. Therefore, we did not test sensors immobilized with higher concentrations of primary anti-*Pf*HRP2 antibody and selected 100 µg/mL as the optimal concentration.

### *Pf*HRP2 detection in whole blood samples

Blood contains several electroactive species (glucose, uric acid, ascorbic acid) which can interfere with electrochemical measurements^32^. Of these, ascorbic acid has been shown to generate a significant interference effect^18,19^. To address this issue, AO was incorporated into the Vivid membrane to react with ascorbic acid, causing it to be consumed prior to encountering the electrode. The applied bias potential was also optimized to minimize interference caused by electroactive species in blood. To evaluate the effectiveness of these approaches, chronocoulometric measurements of whole blood samples spiked with interfering species (0.1 mM ascorbic acid, 20 mM glucose, and 0.1 mM uric acid) and non-spiked blood samples containing zero or 50 µg/mL of *Pf*HRP2 were performed using the assembled immunosensor. As shown in Supplementary Fig. S6, there is a negligible difference between the background and detection signals for the spiked and non-spiked samples, which demonstrates that electroactive species in blood do not interfere with this assay.

**Figure 5:**
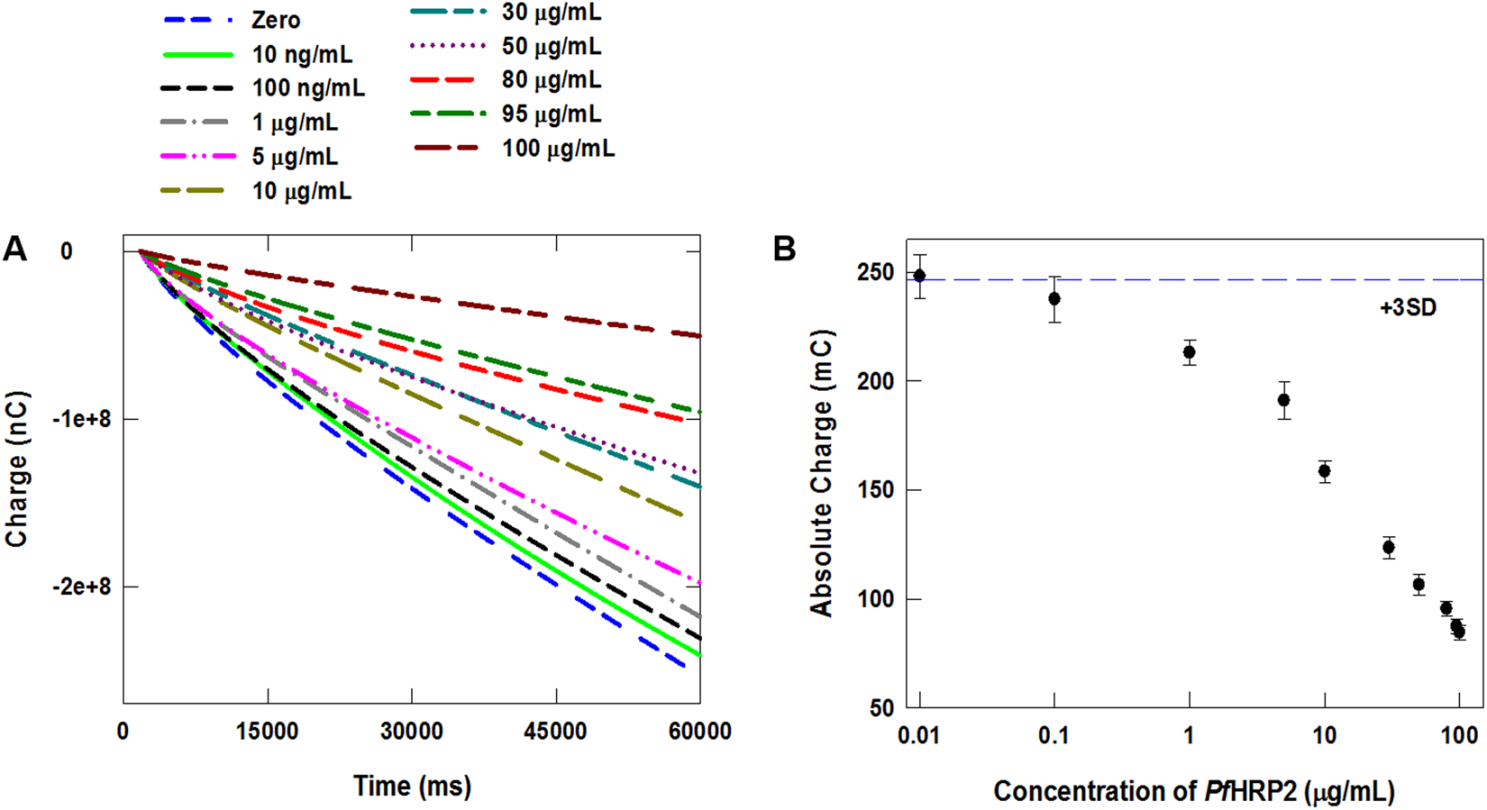
(**A**) Chronocoulograms of whole blood containing various concentrations of *Pf*HRP2 recorded at −0.35 V using the immunosensor. (**B**) Calibration plot based on chronocoulometric charges at 60 sec obtained from the response profile in panel A. Each data point represents the mean ± SD of three separate measurements using new sensors. The dashed line corresponds to 3× the charge SD at zero concentration determined by seven measurements.

To determine the detection range of this immunoassay, measurements were performed using whole blood samples spiked with *Pf*HRP2 from 10 ng/mL to 100 µg/mL. The chronocoulometric response profiles and corresponding calibration plot are shown in Fig. 5A,B, respectively. Based on these results, this wash-free and label-free assay exhibits a detection range from 100 ng/mL to 100 µg/mL in blood, which encompasses the levels found in individuals with *P*. *falciparum* infection^20–23^. While previously reported wash-free and label-free immunosensors can achieve higher sensitivities^16–19^, they utilize different detection schemes that exhibit lower dynamic ranges than required for diagnosing *P*. *falciparum* infection. To further evaluate the performance of this assay for *Pf*HRP2 quantification, we performed a blinded experiment to measure *Pf*HRP2 spiked in blood samples at various concentrations, which was prepared by another researcher in the lab. The measured *Pf*HRP2 concentration was calculated using the calibration plot in Fig. 5B, and plotted *vs*. the prepared concentration. As shown in Supplementary Fig. S7, there is good agreement (correlation coefficient, *R*^*2*^ = 0.9885) between the prepared and measured concentrations for all eight samples, demonstrating the capacity of this immunoassay to measure *Pf*HRP2 in clinical blood samples.

### Evaluating assay specificity and stability

**Figure 6:**
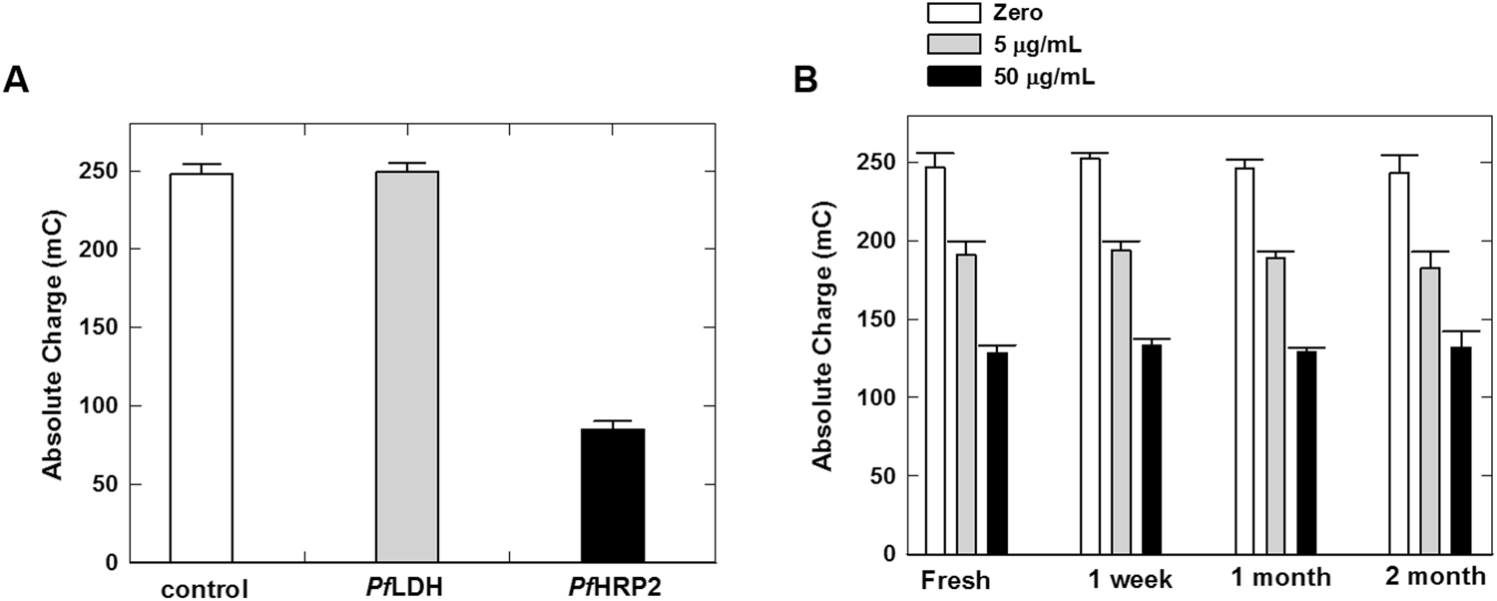
(**A**) Chronocoulometric charges of blood samples containing *Pf*HRP2 (95 µg/mL) or *Pf*LDH (95 µg/mL), and non-spiked blood (blank control). Signals were taken at 60 sec from the chronocoulograms obtained at −0.35 V. Each bar represents the mean ± SD of three separate measurements using new sensors. (**B**) Chronocoulometric charges of blood samples containing zero, 5 µg/mL, or 50 µg/mL of *Pf*HRP2 obtained using fresh sensors and sensors stored at room temperature for varying durations. Each bar represents the mean ± SD of three separate measurements using new sensors.

The specificity of this assay was briefly studied by performing measurements of blood samples spiked with 95 µg/mL of *Pf*HRP2 or *Pf*LDH, another common malaria biomarker, and non-spiked blood. As shown in Fig. 6A, the chronocoulometric signal generated from the blood sample containing *Pf*LDH was similar to the non-spiked blood sample (blank control), indicating that *Pf*LDH will not interfere with this assay. In contrast, the chronocoulometric signal of the blood sample containing *Pf*HRP2 was ~3× lower than that of the *Pf*LDH-containing sample, demonstrating that this immunosensor is highly specific to *Pf*HRP2. We also studied the stability of this sensor by performing measurements of blood samples containing zero, 5 µg/mL or 50 µg/mL of *Pf*HRP2 using fresh sensors and sensors stored at room temperature for 1 week, 1 month or 2 months. As shown in Fig. 6B, there is a negligible difference in the chronocoulometric signals of the fresh and stored sensors at all three concentrations, indicating that this immunosensor exhibits excellent stability at room temperature.

## Conclusion

We present a wash-free and label-free immunoassay for rapid electrochemical measurements of protein biomarkers in whole blood samples. This assay is based on a unique detection scheme involving EC redox cycling for label-free signal amplification and an affinity-based protein quantification strategy where surface attachment of the target antigen to the electrode surface forms an insulator layer that diminishes the electrochemical signal. Compared with other immunoassays that require protein labeling and/or washing, this immunosensor requires only two liquid dispensing steps, and each measurement can be completed in 5 min. Additionally, all of the reagents required for this assay are dried and stored on membranes, enabling simplified operation and excellent stability at room temperature for up to 2 months. Proof-of-concept was carried out by using this immunosensor to measure *Pf*HRP2 in whole blood samples, which could be detected from 100 ng/mL–100 µg/mL, which encompasses the clinically relevant levels found in *P*. *falciparum* infected individuals. Experiments to evaluate the specificity and stability of this immunosensor revealed that it is highly specific to *Pf*HRP2 in whole blood with negligible interference from irrelevant species. While this proof-of-concept assay was designed for *Pf*HPR2 quantification, it would be possible to adapt this technology for multiplexed protein detection, which could provide enhanced diagnostic value for other clinical applications. For example, two or more electrochemical sensors immobilized with capture antibodies specific to other antigens could be fabricated on a single device, and the corresponding detection antibodies could be incorporated onto the stacked membrane assembly. These features, along with its use of low-cost materials (plastics, cellulose membranes), make this a promising platform for point-of-care testing in resource-limited settings.

## Methods and Materials

### Biochemicals and reagents

Hexaamineruthenium (III) chloride (Ru(NH_3_)_6_^3+^), methylene blue, phosphate-buffered saline (PBS, pH 7.4), Traut’s Reagent, (ethylenedinitrilo)tetraacetic acid (EDTA), ascorbate oxidase (AO), casein, and other reagents for buffer solutions were purchased from Sigma-Aldrich (St. Louis, MO). Deionized (DI) water (18.3 MΩ-cm^−1^) was generated using a Smart2Pure water purification system. StabilBlock® Immunoassay Stabilizer was purchased from SurModics, Inc. (Eden Prairie, MN). Mouse monoclonal anti-*Pf*HRP2 IgM and mouse monoclonal anti-*Pf*HRP2 IgG were purchased from ICL, Inc. (Portland, OR). Recombinant *P*. *falciparum* histidine-rich protein 2 (*Pf*HRP2) and *P*. *falciparum* lactate dehydrogenase (*Pf*LDH) were purchased from CTK Biotech (San Diego, CA).

### Thiolation of capture antibody

Anti-*Pf*HRP2 IgM was thiolated (-SH) as previously reported with minor modification^33^. Briefly, 1 mL of 100 µg/mL anti-*Pf*HRP2 IgM was incubated in a solution of Traut’s Reagent in PBS containing 2 mM EDTA for 1 hr at room temperature with gentle agitation. A 10-fold molar excess of Traut’s Reagent per mol antibody was used to ensure full thiolation to the lysine side chains of IgM. Excess (unconjugated) Traut’s Reagent was removed by centrifugation for 30 min at 10,000 rpm. Thiolated anti-*Pf*HRP2 IgM was dissolved in 1 mL of PBS and used immediately for sensor immobilization.

### Sensor fabrication

Au tri-electrode sensors were purchased from GeneFluidics (Irwindale, CA) and membranes were purchased from Pall Corporation (Port Washington, NY). Immobilization of the capture antibody to the working electrode was carried out by incubating 100 µg/mL of thiolated anti-*Pf*HRP2 IgM solution for 1 hr at room temperature followed by thoroughly rinsing with PBS and drying with purified N_2_ gas. To minimize nonspecific binding and enhance the stability of the immobilized antibody, a 30% StabilBlock® Immunoassay Stabilizer solution containing 2% casein in PBS was incubated on the antibody-immobilized electrode for 30 min at room temperature, followed by rinsing twice with PBS and drying with purified N_2_ gas. Circular pieces (7 mm in diameter) of Vivid Plasma Separation membrane and cellulose membranes were cut using a Universal Laser Systems CO_2_ laser cutter. 25 µL of 50 U/mL AO was drop cast on the Vivid membrane, while 10 µL of 1 mM Ru(NH_3_)_6_^3+^ solution and a mixture of 10 µL of 50 µM methylene blue and 100 µg/mL of anti-*Pf*HRP2 IgG were drop cast on separate cellulose membranes. All of the membranes were dried overnight at room temperature inside a desiccator (~30% relative humidity). The immunosensor was assembled by stacking the Vivid and cellulose membranes on top of the Au electrodes, as shown in Fig. 4. Double-sided, pressure-sensitive adhesive (Adhesives Research, Inc., Glen Rock, PA) was cut into rings using a laser cutter and affixed to the top and bottom surfaces of each membrane to secure them to each other and the electrode substrate. A hydrophobic polyethylene terephthalate (PET) film (McMaster-Carr, Elmhurst, IL), containing a 4.5 mm diameter through-hole, was attached to the top of the Vivid membrane to prevent lateral spreading of the liquid sample. The assembled immunosensors were used immediately or stored at room temperature inside a desiccator (~30% relative humidity) for up to 2 months prior to measurements.

### Electrochemical measurements and data analysis

De-identified blood samples from healthy humans were purchased from Bioreclamation Inc. (Westbury, NY). All experimental methods involving blood samples were in accordance with relevant human subjects protection and biosafety guidelines and regulations. *Pf*HRP2 or *Pf*LDH was serially diluted in whole blood and used for electrochemical measurements without any further processing. 10 µL of spiked blood was dispensed onto the sensor followed by 30 µL PBS. Cyclic voltammetric and chronoamperometric measurements were performed after five minutes using a Helios electrochemical workstation (GeneFluidics, Irwindale, CA). Chronoamperometric signals were obtained at a bias potential of −0.35 V and converted to chronocoulometric data by integrating the current using RStudio software (Boston, MA). Coulometric charges are taken at 60 sec of chronocoulometric response profiles and each data point is plotted as the mean ± standard deviation (SD) of three to seven measurements obtained using new sensors.

## Electronic supplementary material

Supplementary Information

## Data Availability

All data generated or analyzed during this study are included in this published article (and its Supplementary Information files).

## References

[1] Sin ML, Mach KE, Wong PK, Liao JC (2014). Advances and challenges in biosensor-based diagnosis of infectious diseases. Expert Rev. Mol. Diagn..

[2] Soper SA (2006). Point-of-care biosensor systems for cancer diagnostics/prognostics. Biosens. Bioelectron..

[3] Wang J (2006). Electrochemical biosensors: towards point-of-care cancer diagnostics. Biosens. Bioelectron..

[4] Wang Y, Xu H, Zhang J, Li G (2008). Electrochemical sensors for clinic analysis. Sensors.

[5] Tian L, Liu L, Li Y, Wei Q, Cao W (2016). Ultrasensitive sandwich-type electrochemical immunosensor based on trimetallic nanocomposite signal amplification strategy for the ultrasensitive detection of CEA. Sci. Rep..

[6] Huang KJ, Niu DJ, Xie WZ, Wang W (2010). A disposable electrochemical immunosensor for carcinoembryonic antigen based on nano-Au/multi-walled carbon nanotubes-chitosans nanocomposite film modified glassy carbon electrode. Anal. Chim. Acta..

[7] Tang H, Chen J, Nie L, Kuang Y, Yao S (2007). A label-free electrochemical immunoassay for carcinoembryonic antigen (CEA) based on gold nanoparticles (AuNPs) and nonconductive polymer film. Biosens. Bioelectron..

[8] Chen X, Jia X, Han J, Ma J, Ma Z (2013). Electrochemical immunosensor for simultaneous detection of multiplex cancer biomarkers based on graphene nanocomposites. Biosens. Bioelectron..

[9] Wu J, Yan F, Tang J, Zhai C, Ju H (2007). A disposable multianalyte electrochemical immunosensor array for automated simultaneous determination of tumor markers. Clin. Chem..

[10] Zhou C (2015). A sensitive label–free amperometric immunosensor for alpha-fetoprotein based on gold nanorods with different aspect ratio. Sci. Rep..

[11] Dutta G, Lillehoj PB (2017). An ultrasensitive enzyme-free electrochemical immunosensor based on redox cycling amplification using methylene blue. Analyst.

[12] Kavosi B, Salimi A, Hallaj R, Moradi F (2015). Ultrasensitive electrochemical immunosensor for PSA biomarker detection in prostate cancer cells using gold nanoparticles/PAMAM dendrimer loaded with enzyme linked aptamer as integrated triple signal amplification strategy. Biosens. Bioelectron..

[13] Okuno J (2006). Label-free immunosensor for prostate-specific antigen based on single-walled carbon nanotube array-modified microelectrodes. Biosens Bioelectron..

[14] Zhang K, Lv S, Lin Z, Li M, Tang D (2018). Bio-bar-code-based photoelectrochemical immunoassay for sensitive detection of prostate-specific antigen using rolling circle amplification and enzymatic biocatalytic precipitation. Biosens Bioelectron.

[15] Iyer PV, Ananthanarayan L (2008). Enzyme stability and stabilization—Aqueous and non-aqueous environment. Process Biochem..

[16] Wang Y (2017). Ultrasensitive label-free electrochemical immunosensor based on multifunctionalized graphene nanocomposites for the detection of alpha fetoprotein. Sci. Rep..

[17] Wang H, Gao X, Ma Z (2017). Multifunctional substrate of label-free electrochemical immunosensor for ultrasensitive detection of cytokeratins antigen 21-1. Sci. Rep..

[18] Dutta G, Kim S, Park S, Yang H (2014). Washing-free heterogeneous immunosensor using proximity-dependent electron mediation between an enzyme label and an electrode. Anal. Chem..

[19] Dutta G (2015). Low-interference washing-free electrochemical immunosensor using glycerol-3-phosphate dehydrogenase as an enzyme label. Anal. Chem..

[20] Hendriksen ICE (2013). Defining falciparum-malaria-attributable severe febrile illness in moderate-to-high transmission settings on the basis of plasma *Pf*HRP2 concentration. J. Infect. Dis..

[21] Hendriksen ICE (2012). Diagnosing severe falciparum malaria in parasitaemic African children: A prospective evaluation of plasma *Pf*HRP2 measurement. PLoS Med..

[22] Seydel KB (2012). Plasma concentrations of parasite histidine-rich protein 2 distinguish between retinopathy-positive and retinopathy-negative cerebral malaria in Malawian children. J. Infect. Dis..

[23] Fox LL (2013). Histidine-rich protein 2 plasma levels predict progression to cerebral malaria in Malawian children with *Plasmodium falciparum* infection. J. Infect. Dis..

[24] Noedl H (2006). Sensitivity and specificity of an antigen detection ELISA for malaria diagnosis. Am. J. Trop. Med. Hyg..

[25] Kifude CM (2008). Enzyme-linked immunosorbent assay for detection of *Plasmodium falciparum* histidine-rich protein 2 in blood, plasma, and serum. Clin. Vaccine Immunol..

[26] Chaorattanakawee S (2013). Direct comparison of the histidine-rich protein-2 enzyme-linked immunosorbent assay (HRP-2 ELISA) and malaria SYBR green I fluorescence (MSF) drug sensitivity tests in *Plasmodium falciparum* reference clones and fresh *ex vivo* field isolates from Cambodia. Malar. J..

[27] Parra ME, Evans CB, Taylor DW (1991). Identification of *Plasmodium falciparum* histidine-rich protein 2 in the plasma of humans with malaria. J. Clin. Microbiol..

[28] Castro-Sesquen YE, Kim C, Gilman RH, Sullivan DJ, Searson PC (2016). Nanoparticle-based histidine-rich protein-2 assay for the detection of the malaria parasite *Plasmodium falciparum*. Am. J. Trop. Med. Hyg.

[29] Laurent A (2010). Performance of HRP-2 based rapid diagnostic test for malaria and its variation with age in an area of intense malaria transmission in southern Tanzania. Malar. J..

[30] Maltha J, Gillet P, Jacobs J (2013). Malaria rapid diagnostic tests in endemic settings. Clin. Microbiol. Infec..

[31] Mouatcho JC, Goldring JP (2013). Malaria rapid diagnostic tests: challenges and Prospects. J. Med. Microbol..

[32] Jia W-Z, Wang K, Xia X-H (2010). Elimination of electrochemical interferences in glucose biosensors. Trends Anal. Chem..

[33] Wang X, Mei Z, Wang Y, Tang L (2015). Gold nanorod biochip functionalization by antibody thiolation. Talanta.

