# Supplementary material for: Wash-free, label-free immunoassay for rapid electrochemical detection of PfHRP2 in whole blood samples
Source: Sci Rep. 2018 Nov 20;8:17129. doi: 10.1038/s41598-018-35471-8 (PMC6244414; doi:10.1038/s41598-018-35471-8)
Supplement: Supplementary file 1 — Supplementary Information [file 41598_2018_35471_MOESM1_ESM.doc]

**Supplementary Information**

**Wash-free, label-free immunoassay for rapid electrochemical detection of *Pf*HRP2 in whole blood samples**

Gorachand Dutta^1,2^ and Peter B. Lillehoj^*3,4^

^1^Department of Electronic and Electrical Engineering, University of Bath, Claverton Down, Bath, BA2 7AY, UK

^2^Centre for Biosensors, Bioelectronics and Biodevices, University of Bath, Claverton Down, Bath, BA2 7AY, UK

^3^Department of Mechanical Engineering, Michigan State University, East Lansing, MI 48824, USA

^4^Department of Biomedical Engineering, Michigan State University, East Lansing, MI 48824, USA

*Corresponding author: Phone: +1-517-432-2976; E-mail: [lillehoj@egr.msu.edu](mailto:lillehoj@egr.msu.edu)

**Fig. S1.** Chronocoulometric signals of blood samples spiked with zero or 50 µg/mL of *Pf*HRP2 obtained from assembled sensors containing 50 µM of methylene blue, 100 µg/mL of secondary anti-*Pf*HRP2 antibody, and varying concentration of Ru(NH_3_)_6_^3+^. Charges are taken at 60 sec of chronocoulograms recorded at ‒0.35 V. Each bar represents the mean ± SD of three separate measurements obtained using new sensors.

**Fig. S2.** Chronocoulometric signals of blood samples spiked with zero or 50 µg/mL of *Pf*HRP2 obtained from assembled sensors containing 1 mM Ru(NH_3_)_6_^3+^, 100 µg/mL of secondary anti-*Pf*HRP2 antibody, and varying concentration of methylene blue (MB). Charges are taken at 60 sec of chronocoulograms recorded at ‒0.35 V. Each bar represents the mean ± SD of three separate measurements obtained using new sensors.

**Fig. S3.** Chronocoulometric signals of blood samples spiked with zero or 50 µg/mL of *Pf*HRP2 obtained from assembled sensors containing 1 mM Ru(NH_3_)_6_^3+^, 100 µg/mL of secondary anti-*Pf*HRP2 antibody, and 50 µM of methylene blue. Charges are taken at 60 sec of chronocoulograms recorded at varying bias potentials. Each bar represents the mean ± SD of three separate measurements obtained using new sensors.

**Fig. S4.** Chronocoulometric signals of blood samples spiked with zero or 50 µg/mL of *Pf*HRP2 obtained from assembled sensors containing 1 mM Ru(NH_3_)_6_^3+^, 50 µM of methylene blue, and varying concentrations of secondary anti-*Pf*HRP2 antibody. Charges are taken at 60 sec of chronocoulograms recorded at ‒0.35 V. Each bar represents the mean ± SD of three separate measurements obtained using new sensors.

**Fig. S5.** Chronocoulometric signals of blood samples spiked with zero or 50 µg/mL of *Pf*HRP2 obtained from assembled sensors containing 1 mM Ru(NH_3_)_6_^3+^, 50 µM of methylene blue, 100 µg/mL of secondary anti-*Pf*HRP2 antibody, and varying concentrations of primary anti-*Pf*HRP2 antibody. Charges are taken at 60 sec of chronocoulograms recorded at ‒0.35 V. Each bar represents the mean ± SD of three separate measurements obtained using new sensors.

**Fig. S6.** Chronocoulometric signals of blood samples containing zero or 50 µg/mL of *Pf*HRP2 spiked with 0.1 mM of ascorbic acid, 20 mM of glucose, and 0.1 mM of uric acid (i), and blood samples not spiked with interfering species (ii). Each bar represents the mean ± SD of three separate measurements obtained using new sensors.

**Fig. S7.** Prepared *Pf*HRP2 concentration *vs*. measured *Pf*HRP2 concentration in whole blood obtained using assembled immunosensors.
